# Clinical outcomes of the eight-chop technique in white cataract: a retrospective case series

**DOI:** 10.1186/s12886-026-04859-w

**Published:** 2026-04-29

**Authors:** Tsuyoshi Sato

**Affiliations:** Ophthalmology, Sato Eye Clinic, 3-3, Nemoto, Matsudo-shi, Chiba-ken 271-0077 Japan

**Keywords:** White cataract, Cataract surgery, Corneal endothelial cell, Eight-chop technique, Phacoemulsification

## Abstract

**Background:**

White cataracts pose substantial technical challenges during phacoemulsification because preoperative assessment of nuclear hardness is impossible and the risk of intraoperative complications is increased. The Eight-chop technique was developed to mechanically fragment the lens nucleus into eight pieces prior to ultrasound emulsification, potentially reducing intraocular stress. This study aimed to evaluate the safety and efficacy of the Eight-chop technique in eyes with white cataract and to compare surgical outcomes according to intraoperatively assessed nuclear hardness.

**Methods:**

This retrospective, single-center, single-surgeon observational study included eyes with white cataract that underwent phacoemulsification using the Eight-chop technique between January 2010 and March 2025. White cataract was defined as complete lens opacification with an absent red reflex. Nuclear hardness was classified intraoperatively using the Emery classification. Intraoperative parameters included operative time, phacoemulsification time, cumulative dissipated energy (CDE), aspiration time, and irrigation fluid volume. Postoperative outcomes included corneal endothelial cell density (CECD), endothelial morphology, intraocular pressure (IOP), and best-corrected visual acuity (BCVA), evaluated preoperatively and at 7 and 19 weeks postoperatively. Outcomes were compared among nuclear hardness groups.

**Results:**

Of 12,642 cataract surgeries performed during the study period, 105 eyes with white cataract were included in the final analysis. Operative time, phacoemulsification time, and CDE increased significantly with increasing nuclear hardness (all *p* < 0.01), whereas aspiration time and irrigation fluid volume did not differ significantly among groups. Mean CECD loss at 7 and 19 weeks postoperatively was minimal, with no significant differences among nuclear hardness groups. Transient postoperative changes in corneal endothelial morphology were observed but resolved by 19 weeks. Postoperative IOP tended to decrease significantly in eyes with higher nuclear hardness. BCVA improved markedly in all groups, and early postoperative differences according to nuclear hardness disappeared over time. Posterior capsule rupture occurred in 2 of 105 eyes, and no cases of dropped nucleus were observed.

**Conclusions:**

The Eight-chop technique was associated with safe and efficient phacoemulsification in eyes with white cataract. By mechanically prefragmenting the lens nucleus into eight segments prior to ultrasound application, the technique may reduce intraocular stress and facilitate stable nuclear management even in eyes with advanced nuclear hardness. This approach was associated with favorable endothelial and visual outcomes with an acceptable complication rate in this retrospective series.

**Supplementary Information:**

The online version contains supplementary material available at 10.1186/s12886-026-04859-w.

## Background

White cataracts represent one of the most technically challenging conditions in modern cataract surgery [1–4]. They are characterized by complete opacification of the crystalline lens with an absent or markedly reduced red reflex, which precludes preoperative visualization of the lens nucleus and posterior capsule. As a result, surgeons must manage increased risks of anterior capsule tear, posterior capsule rupture, zonular instability, and corneal endothelial damage during phacoemulsification [1, 3, 4]. Unlike ordinary cataracts, white cataracts do not allow accurate preoperative assessment of nuclear hardness. Surgical difficulty is therefore determined intraoperatively, often after capsulorrhexis and partial debulking of the lens material. Furthermore, these eyes frequently exhibit elevated intralenticular pressure, fragile capsules, and weakened zonules, all of which contribute to an increased incidence of intraoperative complications [1, 4–6]. Consequently, safe and efficient nucleus management remains a critical issue in the surgical treatment of white cataracts.

Various surgical techniques have been proposed to address the challenges associated with white cataracts, including extracapsular cataract extraction, divide-and-conquer, phaco-chop, and stop-and-chop techniques [3, 4, 7–10]. However, many of these techniques rely on initial phacoemulsification of an undivided hard nucleus, which can transmit substantial stress to the posterior capsule and zonules. This limitation is particularly problematic in white cataracts, where capsular and zonular fragility is frequently encountered [1, 3]. Therefore, an ideal surgical technique for white cataracts should enable reliable nucleus fragmentation while minimizing ultrasound energy, fluid usage, and mechanical stress on intraocular structures. The Eight-chop technique was developed to address these specific challenges by mechanically dividing the lens nucleus into eight small fragments prior to emulsification [11]. Unlike conventional prechop techniques, which typically divide the nucleus into four sections, the Eight-chop technique consistently achieves eight-piece nuclear fragmentation using specialized instruments designed for precise and controlled mechanical splitting. This approach allows subsequent phacoemulsification and aspiration to be performed efficiently at the iris plane with reduced ultrasound energy and fluidics, thereby potentially minimizing corneal endothelial damage and intraoperative complications.

In eyes with white cataracts, successful completion of continuous curvilinear capsulorhexis (CCC) is a critical initial step, which requires adequate staining and visualization of the anterior lens capsule [6, 12]. The use of brilliant blue G (BBG) has been shown to improve capsular visualization and to enable complete capsulorhexis without evidence of ocular toxicity in experimental models [13]. However, previous clinical studies involving eyes with white cataracts have primarily employed trypan blue for anterior capsule staining, and the use of BBG in this setting has not been reported [1, 4]. Thus, the clinical applicability of BBG for anterior capsule staining in eyes with white cataracts remains to be elucidated.

Although the Eight-chop technique has been reported to be effective for hard cataracts [11, 14], evidence regarding its performance specifically in white cataracts remains limited. Moreover, few studies have systematically evaluated intraoperative efficiency, corneal endothelial outcomes, intraocular pressure changes, and visual recovery in white cataracts according to intraoperative nuclear hardness. Given that nuclear hardness is a major determinant of surgical difficulty and postoperative outcomes, such stratified analyses are essential for accurately assessing the clinical value of surgical techniques in this setting. Therefore, the purpose of the present study was to evaluate the safety and efficacy of the Eight-chop technique in eyes with white cataracts by analyzing intraoperative parameters, corneal endothelial cell density and morphology, intraocular pressure, visual outcomes, and surgical complications. Furthermore, outcomes were compared according to intraoperatively assessed nuclear hardness to clarify the applicability of the Eight-chop technique across a wide spectrum of lens hardness in white cataracts.

## Methods

### Ethical considerations

This retrospective, single-center, single-surgeon, single-arm observational study included eyes of patients with white cataracts who underwent phacoemulsification and posterior chamber intraocular lens (IOL) implantation between 4 January 2010 and 25 March 2025. The study was conducted in accordance with the tenets of the Declaration of Helsinki and was approved by the Ethics Committee of Sato Eye Clinic (the approval number: 20091101). Written informed consent was obtained from all patients prior to enrollment. Brilliant Blue G (BBG), which has been approved for human intraocular use in the European Union and is currently under regulatory review by the Ministry of Health, Labour and Welfare in Japan, was used at a concentration of 0.025% for anterior capsule staining in the present study. Because the study was initiated on January 4, 2010, BBG was permitted for use at that time with approval from the institutional review board, which was obtained prior to patient enrollment.

### Study population

Patients who presented to our institution with white cataracts were enrolled in this study. In this study, white cataract was defined as a cataract in which the entire lens appeared white and opaque on slit-lamp examination, with absent or markedly reduced red reflex. Eyes with corneal disease or corneal opacity, uveitis, a history of ocular trauma, or previous intraocular surgery were excluded from the study.

### Preoperative assessment

All patients underwent comprehensive preoperative ophthalmic examinations. The preoperative assessment included slit-lamp biomicroscopy and fundus examination. Best-corrected visual acuity (BCVA) was measured using a Snellen visual acuity chart, and intraocular pressure (IOP) was measured using standard tonometric methods. Corneal endothelial cell density (CECD, cells/mm²), central corneal thickness (CCT), the coefficient of variation in cell size (CV), and the percentage of hexagonal cells (PHC) were evaluated using a non-contact specular microscope (EM-3000; Topcon Corporation, Tokyo, Japan). Axial length and anterior chamber depth were assessed using a swept-source optical coherence tomography biometer with a wavelength of 1060 nm (OA-2000; TOMEY, Tokyo, Japan) when available. In earlier cases, axial length was measured using other commercially available biometry devices, and anterior chamber depth was not consistently assessed in all patients.

### Classification of nuclear hardness

The hardness of the lens nucleus was assessed intraoperatively by the surgeon based on the Emery classification [15]. Because the lens nucleus in white cataracts cannot be evaluated preoperatively, nuclear hardness was classified according to intraoperative findings. After completion of continuous curvilinear capsulorrhexis (CCC) and aspiration of the emulsified lens material, an Eight-chopper was inserted into the exposed lens nucleus, and its color and hardness were evaluated. In eyes with white cataract, removal of the surrounding soft material is necessary to expose the nucleus, and the assessment was based on the intrinsic color and consistency of the nucleus itself. Therefore, this process was not considered to affect the evaluation of nuclear hardness.

### Surgical technique

**Fig. 1:**
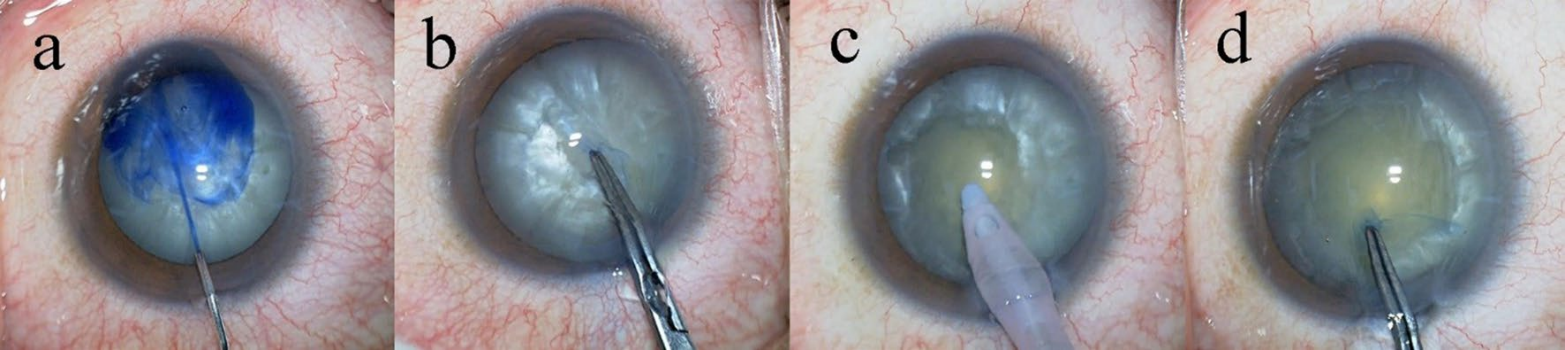
(**a**) Visualization of the anterior lens capsule by staining with 0.025% brilliant blue G. (**b**) Creation of a small continuous curvilinear capsulorhexis (3.0–4.0 mm) using capsulorhexis forceps. (**c**) Aspiration of liquefied lens material through the capsulorhexis to relieve intralenticular pressure and prevent uncontrolled extension of the capsular tear, followed by reformation of the anterior chamber. (**d**) Enlargement of the continuous curvilinear capsulorhexis to a final diameter of 6.2–6.5 mm using capsulorhexis forceps

**Fig. 2:**
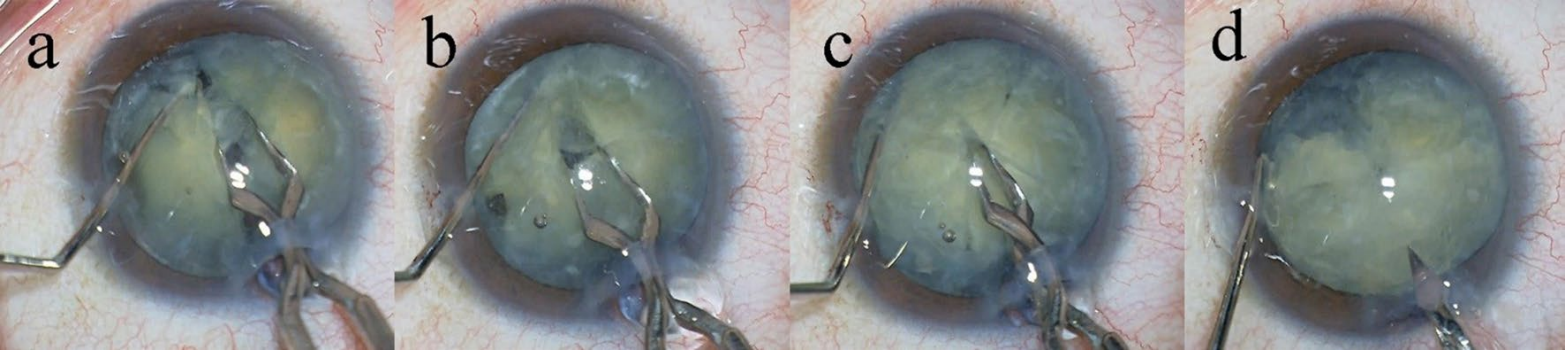
(**a**) Initial division of the lens nucleus into two hemispheres using a Lance chopper, with a nucleus sustainer (AE-2530; ASICO, Westmont, Illinois, USA) inserted through a side-port incision to support the equator of the nucleus. (**b**) Rotation of the nucleus by 90° followed by further division into four quadrants. (**c**) Rotation of the quadranted nucleus by 45° and subsequent division into six segments. (**d**) Final division of the remaining nuclear quadrant to complete segmentation into eight pieces

All surgeries were performed by a single surgeon (T.S.) who was fully experienced in the Eight-chop technique, following the same standardized surgical protocol. Phacoemulsification was performed using the Infiniti^®^ Vision System (Alcon Laboratories, Inc., Fort Worth, TX, USA) in cases prior to 2015 and the Centurion^®^ Vision System (Alcon Laboratories, Inc., Fort Worth, TX, USA) thereafter. All procedures were recorded using a video camera (MKC-704KHD; Ikegami Tsushinki Co., Ltd., Tokyo, Japan). For nucleus division, the Eight-chopper I (SP-8193; ASICO, Westmont, IL, USA) and the Universal prechopper (AE4192; ASICO, Westmont, IL, USA) were used between 2010 and 2018. In 2019, the Lance chopper (SP-9989; ASICO, Westmont, IL, USA) and the Eight-chopper II (SP-8402; ASICO, Westmont, IL, USA) were introduced and subsequently used in combination with the Eight-chopper I. A 3.0-mm temporal clear corneal incision was created using a steel keratome. In all cases, Brilliant Blue G (BBG; 0.025%) was used to enhance visualization of the anterior capsule (Fig. 1). Anterior capsule staining was confirmed by video recordings in each case. After intentionally creating a small CCC, the capsulorrhexis was subsequently enlarged to a final diameter of 6.2–6.5 mm using a two-step CCC technique. In most cases, surgery was performed under the assumption of potential intumescence. A high-viscosity ophthalmic viscosurgical device was injected into the anterior chamber to counteract intralenticular pressure and to prevent uncontrolled peripheral extension of the anterior capsular tear during the two-stage CCC. Hydrodissection was performed using a 27-gauge cannula (AMO Japan, Inc., Tokyo, Japan). The lens nucleus was mechanically divided into eight segments using the Eight-chopper II in 18 cases and the Lance chopper in 87 cases (Fig. 2). In eyes with Grade III or IV nuclear hardness, an additional side-port incision was created using a 23-gauge microvitreoretinal knife at approximately 90° from the main corneal incision, through which a sustainer was inserted. After removal of cortical material from the capsular bag using an irrigation–aspiration tip, a viscoelastic agent was injected, and a foldable posterior chamber intraocular lens (Acrysof^®^ MN60AC; Alcon Laboratories, Inc., Fort Worth, TX, USA) with polymethyl methacrylate haptics was implanted into the capsular bag using an injector system. Residual viscoelastic material was then thoroughly aspirated. At the end of surgery, moxifloxacin (0.5 mg/mL) was injected into the anterior chamber.

### Postoperative examinations

Intraoperative outcome measures included surgical time (minutes), ultrasound time (seconds), aspiration time (minutes), cumulative dissipated energy (CDE), total volume of irrigation fluid used (mL), and the incidence of intraoperative complications. Surgical time was defined as the duration from the initiation of the corneal incision to the completion of viscoelastic material removal. Postoperative examinations were performed on postoperative days 1 and 2, and at 1, 3, 7, and 19 weeks after surgery. Postoperative outcome measures included best-corrected visual acuity, intraocular pressure, and corneal endothelial cell density. For the purposes of analysis in this study, data obtained at 7 and 19 weeks postoperatively were used.

### Handling of missing data and bilateral cases

Because this was a retrospective study, postoperative data were missing for some eyes. The primary reasons for missing data were loss to follow-up or the inability to obtain corneal endothelial cell measurements. Therefore, statistical analyses were performed using a complete-case analysis approach, and no imputation of missing values was conducted. A total of 12 eyes were derived from bilateral cases. These eyes accounted for a small proportion of the overall study population, and no patient contributed more than two eyes to the dataset. Given the limited number of bilateral cases and the predominance of unilateral observations, the potential impact of inter-eye correlation on the overall estimates was considered minimal. Therefore, no statistical adjustment for within-subject correlation, such as the use of mixed-effects models or generalized estimating equations, was applied in the analyses.

### Statistical analysis

Statistical analyses were performed using R software (R version 4.5.2; R Foundation for Statistical Computing, Vienna, Austria). Continuous variables are presented as mean ± standard deviation. Categorical variables are presented as counts and percentages. Comparisons among groups stratified by nuclear hardness were performed using one-way analysis of variance (ANOVA) for continuous variables. When overall significance was detected, post hoc pairwise comparisons were conducted using the Tukey–Kramer method. For comparisons of categorical variables, the chi-square test or Fisher’s exact test was applied, as appropriate. BCVA was analyzed using the Kruskal–Wallis test because its distribution did not meet the assumptions of normality. Longitudinal changes in postoperative outcomes were evaluated by comparing values at each postoperative time point using appropriate statistical tests. Because this was a retrospective study and postoperative data were missing for some eyes, all analyses were conducted using a complete-case analysis approach without imputation of missing values. A p-value < 0.05 was considered statistically significant. All statistical tests were two-tailed.

## Results

During the observation period from January 4, 2010, to March 25, 2025, a total of 12,642 cataract surgeries were performed at our institution. Among these, 126 eyes were diagnosed with white cataracts. Of the 126 eyes, 20 eyes were excluded due to loss to follow-up, and 1 eye was excluded because of progression to lens dislocation. Consequently, data from 105 eyes were included in the final analysis. Among the 105 eyes with white cataracts, 22 eyes were from patients with diabetes mellitus. Postoperatively, 4 eyes were excluded from visual acuity analyses due to diabetic retinopathy, 3 eyes due to age-related macular degeneration, and 1 eye due to branch retinal vein occlusion. All other missing data were attributable to lack of patient follow-up or inability to perform the required examinations.

### Preoperative characteristics and intraoperative parameters by lens hardness

Preoperative patient characteristics and intraoperative parameters are summarized according to lens hardness (Table 1). The number of eyes was 13 in the Grade I–II group, 28 in the Grade III group, and 64 in the Grade IV group. The mean age increased significantly with lens hardness, being 44.4 ± 12.0 years in the Grade I–II group, 60.1 ± 11.5 years in the Grade III group, and 73.1 ± 9.0 years in the Grade IV group (*p* < 0.001). There was no significant difference in sex distribution among the groups (male: 61.5%, 67.9%, and 46.9%, respectively; *p* = 0.152). Anterior chamber depth (ACD) was not compared among groups, as data were available for only one eye in the Grade I–II group. Axial length, in contrast, decreased significantly with increasing lens hardness (Grade I–II: 24.7 ± 0.9 mm; Grade III: 24.1 ± 1.2 mm; Grade IV: 23.1 ± 1.0 mm; *p* < 0.001). Lens hardness itself differed significantly among the groups (1.3 ± 0.5, 3.3 ± 0.3, and 4.3 ± 0.3, respectively; *p* < 0.001). Regarding intraoperative parameters, both operative time and phacoemulsification time (Phaco time) increased significantly with lens hardness (operative time: 8.9 ± 4.3, 10.1 ± 4.4, 12.8 ± 4.4 min; *p* = 0.003; Phaco time: 5.8 ± 6.5, 18.9 ± 13, 34 ± 14.6 s; *p* < 0.001). Aspiration time and volume of fluid used did not differ significantly among the groups (*p* = 0.108 and *p* = 0.32, respectively). Cumulative Dissipated Energy (CDE) increased significantly with lens hardness (1.6 ± 2, 7.4 ± 5.5, 15.3 ± 6.4, respectively; *p* < 0.001).

**Table 1:** Preoperative characteristics and intraoperative parameters by Lens hardness

Characteristics/Parameters	Grade I-II	Grade III	Grade IV	*p*-value
Number of eyes	13	28	64	NA
Age (years)	44.4 ± 12.0	60.1 ± 11.5	73.1 ± 9.0	< 0.001
Sex: Male	8 (61.5%)	19 (67.9%)	30 (46.9%)	0.152
Sex: Female	5 (38.5%)	9 (32.1%)	34 (53.1%)	0.152
ACD (mm)	2.5 *	3.2 ± 0.5	3.2 ± 0.6	NA
Axial length (mm)	24.7 ± 0.9	24.1 ± 1.2	23.1 ± 1.0	< 0.001
Lens hardness	1.3 ± 0.5	3.3 ± 0.3	4.3 ± 0.3	< 0.001
Operative time (min)	8.9 ± 4.3	10.1 ± 4.4	12.8 ± 4.4	0.003
Phaco time (s)	5.8 ± 6.5	18.9 ± 13	34 ± 14.6	< 0.001
Aspiration time (s)	128.2 ± 60.5	140.4 ± 35.1	154 ± 44.5	0.108
CDE	1.64 ± 2.02	7.42 ± 5.46	15.30 ± 6.37	< 0.001
Volume of fluid used (mL)	46.8 ± 22.6	57.2 ± 12.4	74 ± 87.2	0.32

NA: Not applicable. *Anterior chamber depth was available for only one eye in the Grade I–II group; therefore, SD could not be calculated

### Corneal endothelial cell density changes by lens hardness

Preoperative and postoperative corneal endothelial cell density (CECD) values are shown according to lens hardness (Table 2). Preoperatively, the mean CECD was 2585.9 ± 403.6 cells/mm² in the Grade I–II group, 2676 ± 180.4 cells/mm² in the Grade III group, and 2627.2 ± 269.6 cells/mm² in the Grade IV group, with no significant difference among the groups (*p* = 0.593). At 7 weeks postoperatively, CECD values were 2491.8 ± 322.3, 2598.8 ± 241.5, and 2421.6 ± 489.3 cells/mm² in the Grade I–II, III, and IV groups, respectively. The mean percentage decrease from preoperative values was − 2.7 ± 11.5% in Grade I–II, -1.6 ± 7.1% in Grade III, and − 6.4 ± 17.4% in Grade IV, with no significant difference among the groups (*p* = 0.242 for CECD, *p* = 0.389 for % decrease). At 19 weeks postoperatively, CECD values were 2539.3 ± 110.6, 2629.1 ± 235.6, and 2433.4 ± 418.1 cells/mm², with mean percentage decreases of -0.9 ± 6.8%, -0.5 ± 5.7%, and − 6.7 ± 13.5% in the Grade I–II, III, and IV groups, respectively. No statistically significant differences were observed among the groups (*p* = 0.238 for CECD, *p* = 0.105 for % decrease). In the Grade IV group, the mean percentage decrease in CECD at 7 weeks was − 6.4% (95% CI, -11.4 to -1.5).

**Table 2:** Pre- and postoperative CECD values

Time Period	Mean CECD ± SD and % Decrease			
	Grade I-II (*n* = 13)	Grade III (*n* = 28)	Grade IV (*n* = 64)	*p*-value
Preoperatively	2585.9 ± 403.6	2676.0 ± 180.4	2627.2 ± 269.6	0.593
7 weeks postoperatively	2491.8 ± 322.3	2598.8 ± 241.5	2421.6 ± 489.3	0.242
% Decrease	-2.7 ± 11.5	-1.6 ± 7.1	-6.4 ± 17.4	0.389
19 weeks postoperatively	2539.3 ± 110.6	2629.1 ± 235.6	2433.4 ± 418.1	0.238
% Decrease	-0.9 ± 6.8	-0.5 ± 5.7	-6.7 ± 13.5	0.105

### Corneal endothelial parameters according to lens hardness

The pre- and postoperative central corneal thickness (CCT), coefficient of variation of cell size (CV), and percentage of hexagonal cells (PHC) according to lens hardness are shown in Table 3. Preoperatively, CCT was 550.0 ± 46.2 μm in the Grade I–II group, 544.0 ± 49.0 μm in the Grade III group, and 541.1 ± 41.9 μm in the Grade IV group, with no significant differences among the groups (*p* = 0.820). At 7 weeks postoperatively, CCT was 551.8 ± 45.2, 563.2 ± 60.9, and 552.3 ± 56.6 μm, and at 19 weeks postoperatively, 518.3 ± 20.9, 536.2 ± 44.2, and 542.6 ± 45.7 μm, with no significant differences among the groups at either time point. Preoperative CV was 48.3 ± 9.6 in the Grade I–II group, 39.8 ± 4.9 in the Grade III group, and 44.3 ± 10.7 in the Grade IV group, showing a significant difference among groups (*p* = 0.023). At 7 weeks postoperatively, CV was 50.4 ± 10.3, 39.9 ± 5.7, and 43.9 ± 8.2, with significant differences (*p* = 0.001), whereas at 19 weeks postoperatively, CV was 41.6 ± 5.7, 39.7 ± 8.1, and 39.5 ± 5.7, and the differences were no longer significant (*p* = 0.686). Preoperative PHC was 39.0 ± 10.6% in the Grade I–II group, 44.3 ± 8.3% in the Grade III group, and 42.8 ± 7.8% in the Grade IV group, with no significant differences among groups (*p* = 0.511). At 7 weeks postoperatively, PHC was 39.0 ± 8.3%, 45.2 ± 8.9%, and 41.3 ± 8.3%, and at 19 weeks postoperatively, 39.3 ± 7.2%, 46.6 ± 7.8%, and 43.4 ± 7.9%, with no significant differences at either time point.

**Table 3:** Pre- and postoperative endothelial CCT, CV, and PHC by Lens hardness

Variable	Time period	Grade I-II (*n* = 13)	Grade III (*n* = 28)	Grade IV (*n* = 64)	*p*-value
CCT (Mean ± SD)	Preoperative	550.0 ± 46.2	544.0 ± 49.0	541.1 ± 41.9	0.820
	7 weeks postoperatively	551.8 ± 45.2	563.2 ± 60.9	552.3 ± 56.6	0.731
	19 weeks postoperatively	518.3 ± 20.9	536.2 ± 44.2	542.6 ± 45.7	0.403
CV (Mean ± SD)	Preoperative	48.3 ± 9.6	39.8 ± 4.9	44.3 ± 10.7	0.023
	7 weeks postoperatively	50.4 ± 10.3	39.9 ± 5.7	43.9 ± 8.2	0.001
	19 weeks postoperatively	41.6 ± 5.7	39.7 ± 8.1	39.5 ± 5.7	0.686
PHC (Mean ± SD)	Preoperative	39.0 ± 10.6	44.3 ± 8.3	42.8 ± 7.8	0.511
	7 weeks postoperatively	39.0 ± 8.3	45.2 ± 8.9	41.3 ± 8.3	0.217
	19 weeks postoperatively	39.3 ± 7.2	46.6 ± 7.8	43.4 ± 7.9	0.861

Values are presented as mean ± standard deviation (SD). P-values were calculated using one-way analysis of variance (ANOVA)

### Intraocular pressure changes according to lens hardness

**Fig. 3:**
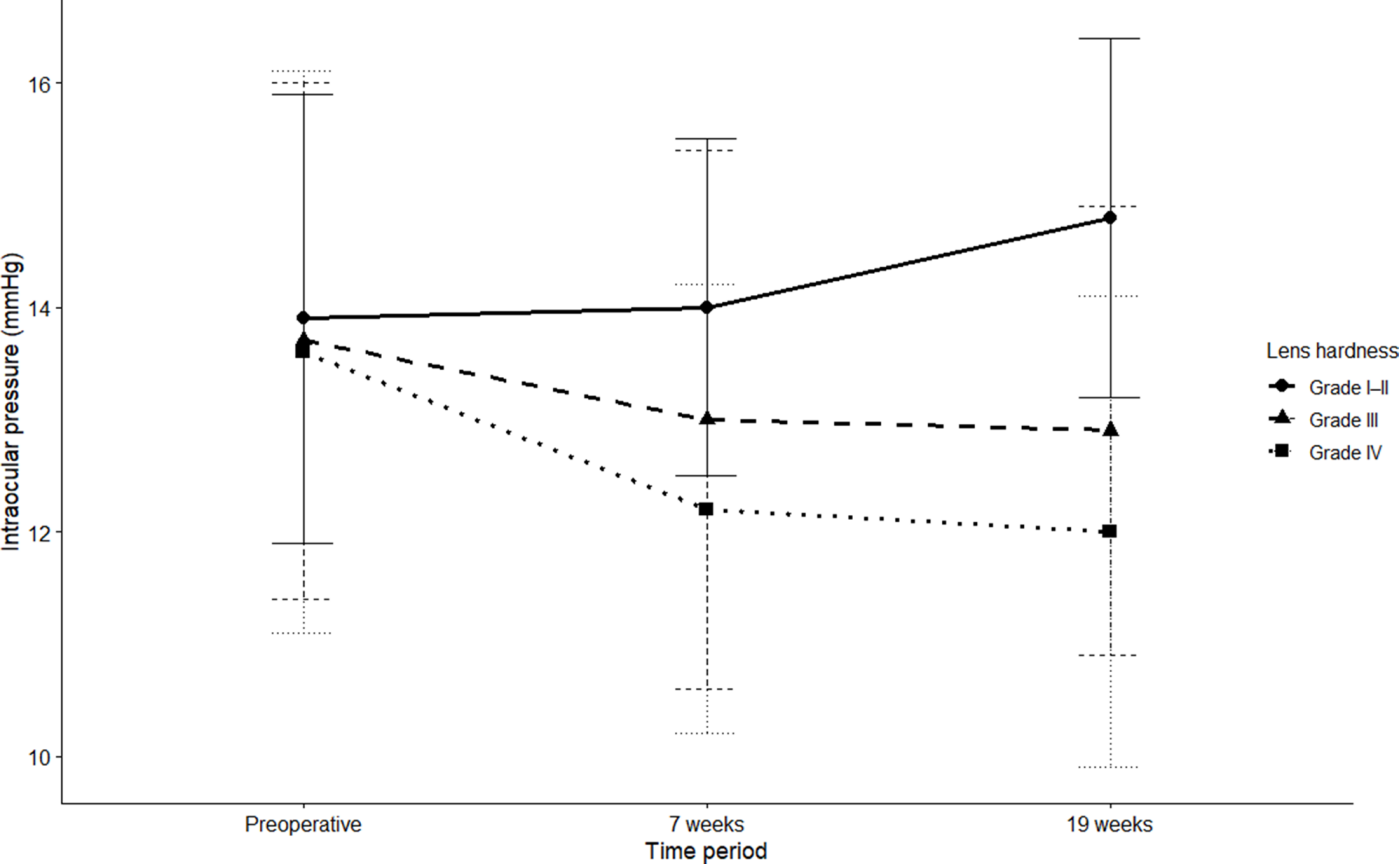
Changes in IOP according to lens hardness. Mean IOP values (mmHg) are plotted over time for lens hardness Grades I–II, III, and IV. p-values for intergroup comparisons at each time point are shown in Table 4

The mean intraocular pressure (IOP) and percentage decrease from baseline according to lens hardness are shown in Table 4. A positive value indicates a decrease in IOP, whereas a negative value indicates an increase relative to baseline. Preoperatively, the mean IOP was 13.9 ± 2.0 mmHg in the Grade I–II group, 13.7 ± 2.3 mmHg in the Grade III group, and 13.6 ± 2.5 mmHg in the Grade IV group, with no significant differences among the groups (*p* = 0.948). At 7 weeks postoperatively, the mean IOP was 14.0 ± 1.5 mmHg, 13.0 ± 2.4 mmHg, and 12.2 ± 2.0 mmHg, with significant differences among the groups (*p* = 0.018). The percentage decrease from baseline IOP was − 3.0 ± 11.6% in the Grade I–II group, 4.8 ± 12.0% in the Grade III group, and 9.7 ± 14.0% in the Grade IV group, showing a significant difference (*p* = 0.013). At 19 weeks postoperatively, mean IOP was 14.8 ± 1.6 mmHg, 12.9 ± 2.0 mmHg, and 12.0 ± 2.1 mmHg (*p* = 0.003), and the percentage decrease from baseline was − 7.5 ± 13.3%, 5.5 ± 7.6%, and 10.0 ± 15.3%, respectively, with significant differences among groups (*p* = 0.007). These results indicate that higher lens hardness tended to be associated with greater postoperative IOP reduction. At 19 weeks postoperatively, the mean decrease in IOP in the Grade IV group was 10.0% (95% CI, 4.8 to 15.2). The changes in intraocular pressure over time are illustrated in Fig. 3.

**Table 4:** Mean IOP (mmHg) and mean decrease (%) over time by lens hardness

Time period	Grade I-II (*n* = 13)	Grade III (*n* = 28)	Grade IV (*n* = 64)	*p*-value
Preoperative IOP (mmHg)	13.9 ± 2.0	13.7 ± 2.3	13.6 ± 2.5	0.948
7 weeks postoperatively	14.0 ± 1.5	13.0 ± 2.4	12.2 ± 2.0	0.018
% Decrease at 7 weeks	-3.0 ± 11.6	4.8 ± 12.0	9.7 ± 14.0	0.013
19 weeks postoperatively	14.8 ± 1.6	12.9 ± 2.0	12.0 ± 2.1	0.003
% Decrease at 19 weeks	-7.5 ± 13.3	5.5 ± 7.6	10.0 ± 15.3	0.007

Negative values indicate an increase in intraocular pressure relative to baseline

### Best-corrected visual acuity according to lens hardness

**Fig. 4:**
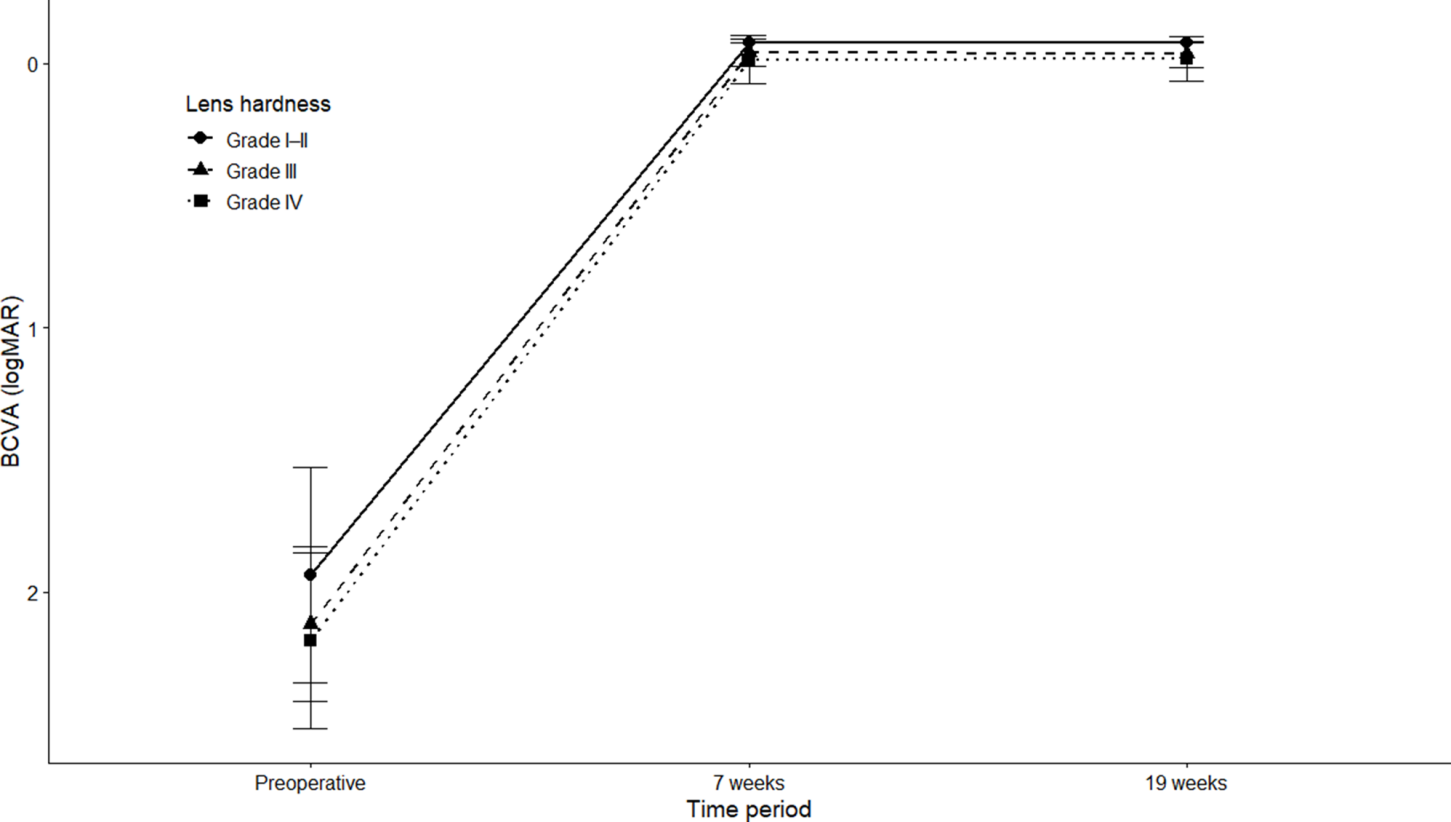
Changes in BCVA over time according to nuclear hardness. BCVA expressed in logMAR units at the preoperative visit and at 7 and 19 weeks postoperatively in eyes with white cataracts, stratified by nuclear hardness (Grade I–II, Grade III, and Grade IV). BCVA improved markedly in all groups after surgery. Although a significant intergroup difference was observed at 7 weeks postoperatively, this difference was not maintained at 19 weeks

The pre- and postoperative best-corrected visual acuity (BCVA) in logMAR according to lens hardness are shown in Table 5. Preoperatively, BCVA was 1.94 ± 0.41 in the Grade I–II group, 2.12 ± 0.29 in the Grade III group, and 2.18 ± 0.33 in the Grade IV group, with no significant differences among the groups (*p* = 0.107). At 7 weeks postoperatively, BCVA was − 0.08 ± 0.00, -0.04 ± 0.05, and − 0.01 ± 0.09, showing a significant difference among groups (*p* = 0.022). Post hoc analysis revealed that BCVA in the Grade I–II group was significantly better than in both the Grade III group (*p* = 0.044) and the Grade IV group (*p* = 0.018), whereas no significant difference was observed between the Grade III and Grade IV groups (*p* = 1.000). At 19 weeks postoperatively, BCVA was − 0.07 ± 0.03, -0.04 ± 0.05, and − 0.02 ± 0.08, with no significant differences among groups (*p* = 0.161). These results suggest that differences in BCVA according to lens hardness were observed in the early postoperative period but tended to disappear over the long term. The changes in BCVA over time are shown in Fig. 4.

**Table 5:** Pre- and postoperative BCVA values over time by lens hardness

Time period	BCVA (logMAR)			*p*-value
	Grade I-II	Grade III	Grade IV	
Preoperatively	1.94 ± 0.41 (*n* = 13)	2.12 ± 0.29 (*n* = 28)	2.18 ± 0.33 (*n* = 64)	0.107
7 weeks postoperatively	-0.08 ± 0.00 (*n* = 11)	-0.04 ± 0.05 (*n* = 24)	-0.01 ± 0.09 (*n* = 47)	0.022
19 weeks postoperatively	-0.07 ± 0.03 (*n* = 8)	-0.04 ± 0.05 (*n* = 13)	-0.02 ± 0.08 (*n* = 31)	0.161

Data are presented as mean ± SD (logMAR). Between-group comparisons were performed using the Kruskal–Wallis test. p-values smaller than 0.001 are reported as *p* < 0.001

### Complications and additional procedures during surgery

In this study, posterior capsule rupture occurred in 2 of 105 eyes. In 7 eyes, a complete CCC could not be achieved. Iris retractor hooks were used in 25 eyes. No cases of lens nucleus drop occurred during surgery.

## Discussion

White cataract represents one of the most technically demanding conditions in phacoemulsification surgery because of the absence of a red reflex, increased intralenticular pressure, frequent liquefaction of cortical material, and the presence of a hard and brittle lens nucleus [1–7]. These characteristics increase the risk of capsulorhexis-related complications, zonular stress, posterior capsule rupture, and excessive ultrasound energy delivery [1, 3, 4]. In this retrospective single-surgeon study, we evaluated the intraoperative efficiency and postoperative outcomes of the Eight-chop technique in eyes with white cataract and demonstrated that this approach was associated with safe and efficient phacoemulsification with favorable visual and endothelial outcomes. In the present study, the Eight-chop technique achieved short operative time, reduced ultrasound exposure, low cumulative dissipated energy (CDE), and limited irrigation fluid usage, even in eyes with advanced nuclear hardness [4, 16]. Surgical efficiency is particularly important in white cataract because prolonged manipulation and excessive energy delivery are known to increase the risk of endothelial damage and intraocular complications [1, 2, 10, 17, 18]. Although direct comparisons of operative time among studies are difficult due to differences in surgical definitions and reporting, the operative time observed in this study was comparable to or shorter than those reported in previous studies using alternative techniques for dense cataracts [8, 10]. Importantly, the Eight-chop technique separates nuclear fragmentation from phacoemulsification and aspiration. This conceptual separation allows the surgeon to complete nuclear division mechanically before ultrasound application, thereby minimizing the need for prolonged phaco power [11]. In contrast, techniques such as divide-and-conquer or phaco-chop require simultaneous nuclear cracking and emulsification, which may increase intraocular stress, particularly in eyes with fragile capsules and weakened zonules, as frequently encountered in white cataract [2, 3, 7].

CECD is widely recognized as an integrated indicator of intraocular surgical stress [19–23]. Previous reports have shown that phacoemulsification in dense or white cataracts is associated with greater endothelial cell loss than routine cataract surgery [1, 2, 10, 16]. Reported endothelial cell loss rates in dense cataracts vary widely, reflecting differences in surgical technique, ultrasound parameters, and case complexity. In the present study, postoperative endothelial cell loss was minimal and not statistically significant compared with baseline values. This finding suggests that the Eight-chop technique was associated with low ultrasound energy delivery and may reduce mechanical and fluidic stress on the corneal endothelium. This energy efficiency is largely attributable to the complete prefragmentation of the lens nucleus into eight small segments before phacoemulsification, which avoids the need for nuclear division during phacoemulsification. In addition, the wedge-induced fracture mechanism allows consistent nuclear splitting irrespective of nuclear hardness, enabling stable and efficient nuclear management across all hardness grades. The ability to divide the nucleus into eight small, manageable segments facilitates rapid emulsification at the iris plane, thereby increasing the distance between the ultrasound tip and the corneal endothelium. Another factor contributing to endothelial safety is the reduced volume of irrigation fluid used during surgery. Excessive fluid flow can induce turbulence within the anterior chamber, leading to endothelial trauma [21, 24–26]. The low fluid usage observed in this study reflects the efficiency of the Eight-chop technique and may partially explain the favorable endothelial outcomes.

In the present study, brilliant blue G (BBG) at a concentration of 0.025% was used routinely for anterior capsule staining in eyes with white cataracts. To the best of our knowledge, this is the first clinical study to apply BBG in this context, as previous studies of white cataracts have relied exclusively on trypan blue for visualization of the anterior capsule [1, 27, 28]. BBG has been extensively studied as a protein-staining dye and has demonstrated a favorable safety profile compared with trypan blue and indocyanine green in experimental and clinical settings [13, 29–31]. Previous experimental studies have shown that this concentration provides sufficient anterior capsule staining with minimal toxicity to corneal endothelial cells, and clinical studies in vitreoretinal surgery have further supported its relative safety [13, 29, 30]. In the present study, BBG provided adequate capsular visualization to achieve complete continuous curvilinear capsulorhexis without intraoperative complications attributable to dye toxicity. The stable postoperative endothelial cell counts observed are consistent with previous findings and support the safety and clinical utility of BBG for anterior capsule staining in white cataract surgery.

Postoperative best-corrected visual acuity (BCVA) improved significantly across all nuclear hardness grades. Although eyes with harder nuclei tended to show slightly delayed visual recovery, BCVA at later postoperative time points did not differ significantly among groups. These results indicate that the Eight-chop technique provides consistent visual rehabilitation even in eyes with severe lens opacification. Several eyes were excluded from visual acuity analysis due to coexisting retinal diseases, including diabetic retinopathy and age-related macular degeneration. This exclusion allowed a more accurate assessment of surgery-related visual outcomes and strengthens the interpretation that postoperative visual improvement was primarily attributable to the surgical technique rather than retinal pathology.

A reduction in IOP following cataract surgery has been reported in both glaucomatous and nonglaucomatous eyes [32, 33]. In the present study, postoperative changes in IOP varied according to nuclear hardness. While a slight increase in IOP was observed in eyes with Grade I–II nuclei, a decrease in IOP was noted in eyes with Grade III and IV nuclei, resulting in significant intergroup differences in postoperative IOP. Although the limited sample size may have reduced the statistical power to detect small changes in IOP, a trend toward IOP reduction was observed in the Grade III and IV groups. These findings suggest that postoperative IOP reduction was observed in association with greater nuclear hardness; however, this potential effect should be confirmed in future prospective studies.

Despite the technical challenges inherent to white cataract surgery, intraoperative complications were infrequent in this study. Posterior capsule rupture occurred in only two eyes, and no cases of nuclear drop were observed. Although incomplete capsulorhexis was encountered in several cases, the two-step capsulorhexis technique combined with BBG staining allowed controlled enlargement of the capsular opening without progression to severe complications. The use of iris retractor hooks in selected cases facilitated adequate surgical exposure and contributed to intraoperative safety [34]. These findings highlight the importance of appropriate adjunctive techniques when managing white cataracts.

Compared with conventional techniques, the Eight-chop method offers several theoretical and practical advantages in white cataract surgery. By enabling complete mechanical segmentation of the nucleus prior to emulsification, it minimizes zonular stress and posterior capsule movement [14, 35]. The use of specialized instruments, such as the Eight-chopper II and Lance chopper, allows precise and controlled nuclear division even in extremely hard nuclei [11]. Furthermore, dividing the nucleus into eight segments reduces the size of each fragment, facilitating efficient aspiration with minimal ultrasound power. This feature is particularly advantageous in eyes with compromised corneal endothelium or shallow anterior chambers [36].

This study has several limitations. First, the single-arm design without a control group precludes direct comparison with other surgical techniques. Therefore, the findings of this study should be interpreted as descriptive observations rather than evidence of superiority. Further comparative studies are required to determine the relative advantages of the Eight-chop technique. Second, all procedures were performed by a single experienced surgeon, which may limit the generalizability of the findings. The learning curve and reproducibility of the Eight-chop technique were not evaluated in this study, and the results may reflect the surgeon’s high level of expertise. Further studies involving multiple surgeons are required to assess the generalizability and reproducibility of this technique. Furthermore, because this study spanned a long period, changes in surgical equipment, instruments, and the surgeon’s experience over time may have influenced the outcomes and should be considered when interpreting the results. In addition, the definition of operative time is not standardized across studies in cataract surgery, which may limit direct comparisons with previously reported results. In the present study, operative time was defined to reflect the overall surgical workflow; however, this difference in definition should be considered when interpreting the findings. Third, the retrospective design may introduce selection bias. In addition, the exclusion of cases due to loss to follow-up may have introduced attrition bias, as patients with missing postoperative data may have had different clinical outcomes, including potentially worse prognosis. The relatively small sample size in the Grade I–II group may have limited the statistical power to detect differences among groups, and therefore the results should be interpreted with caution. Fourth, although a small number of eyes from bilateral cases were included, no adjustment for inter-eye correlation was performed. Although the proportion of such cases was small, this may have influenced the results and should be considered when interpreting the findings. In addition, nuclear hardness was classified based on intraoperative subjective assessment using the Emery classification. This may introduce variability and potential classification bias, and therefore subgroup analyses based on nuclear hardness should be interpreted with caution. A significant difference in baseline CV was also observed among the groups, which may have affected the postoperative comparisons of endothelial morphology and should be considered when interpreting these results. In addition, the relatively large standard deviations observed in some baseline parameters likely reflect heterogeneity in the study population and should also be taken into account.

## Conclusions

In conclusion, the Eight-chop technique was associated with safe and efficient phacoemulsification in eyes with white cataract. The technique achieves favorable intraoperative efficiency, preserves corneal endothelial integrity, and provides favorable visual outcomes with a low rate of complications. These findings support the Eight-chop technique as a valuable surgical option for the management of white cataract, particularly in cases with hard nuclei and increased surgical risk.

## Supplementary Information

Below is the link to the electronic supplementary material.

Supplementary Material 1

## Data Availability

The anonymized dataset generated and analyzed during the present study is available in the Supplementary Information (Supplementary Dataset 1). No personally identifiable information is included, in accordance with ICMJE data sharing standards.

## Abbreviations

IOL: Intraocular lens
BBG: Brilliant Blue G
BCVA: Best-corrected visual acuity
IOP: Intraocular pressure
CECD: Corneal endothelial cell density
CCT: Central corneal thickness
CV: Coefficient of variation
PHC: Percentage of hexagonal cells
ACD: Anterior chamber depth
CDE: Cumulative dissipated energy
ANOVA: Analysis of variance
CCC: Continuous curvilinear capsulorhexis
logMAR: Logarithm of the minimum angle of resolution
